# Deletion of *14-3-3σ* sensitizes mice to DMBA/TPA-induced papillomatosis

**DOI:** 10.18632/oncotarget.10478

**Published:** 2016-07-07

**Authors:** Markus Winter, Dmitri Lodygin, Berlinda Verdoodt, Heiko Hermeking

**Affiliations:** ^1^ Experimental and Molecular Pathology, Institute of Pathology, Ludwig-Maximilians-Universität München, Munich, Germany; ^2^ Institute of Neuroimmunology and Institute for Multiple Sclerosis Research, University Medical Center Göttingen, Göttingen, Germany; ^3^ Institute of Pathology, Ruhr-University Bochum, Bochum, Germany; ^4^ German Cancer Consortium (DKTK), Heidelberg, Germany; ^5^ German Cancer Research Center (DKFZ), Heidelberg, Germany

**Keywords:** 14-3-3sigma knock-out mouse, skin carcinogenesis, repeated epilation/ER

## Abstract

The p53-inducible cell cycle regulator 14-3-3σ exhibits tumor suppressive functions and is highly expressed in differentiating layers of the epidermis and hair follicles. *14-3-3σ/SFN/stratifin* is frequently silenced in human epithelial cancers, and experimental down-regulation of *14-3-3σ* expression immortalizes primary human keratinocytes. In the *repeated-epilation* (*ER*) mouse model, a heterozygous nonsense mutation of *14-3-3σ* causes repeated hair-loss, hyper-proliferative epidermis, and spontaneous development of papillomas and squamous cell carcinomas in aging mice. Therefore, loss of 14-3-3*σ* function might contribute to epithelial tumor development. Here, we generated mice with *loxP* sites surrounding the single *14-3-3σ* exon which allowed Cre-mediated deletion of the gene. *14-3-3σ*-deficient mice are viable, but demonstrate a permanently disheveled fur. However, histological analyses of the skin did not reveal obvious defects in the hair follicles or the epidermis. Deletion of *14-3-3σ* did not enhance spontaneous epidermal tumor development, whereas it increased the frequency and size of DMBA/TPA-induced papillomas. In conclusion, 14-3-3*σ* is dispensable for normal epidermal homeostasis but critical for suppression of chemically-induced skin carcinogenesis. In addition, these results suggest that the *ER* mutation of *14-3-3σ* is not equivalent to loss of 14-3-3*σ*, but may represent a gain-of-function variant, which does not reflect the organismal function of wild-type 14-3-3*σ*.

## INTRODUCTION

The mammalian epidermis undergoes continuous self-renewal to repair damaged tissue and replace aged cells. A single layer of proliferative keratinocytes at the inner base of the epidermis gives rise to non-proliferative keratinocytes which renew the suprabasal (outer) layers, until they reach the outermost layer where they terminally differentiate and are shed [1].

In the human epidermis 14-3-3σ expression gradually increases along the axis of keratinocyte differentiation. Suppression of 14-3-3*σ* expression abrogates terminal keratinocyte differentiation and immortalizes human primary keratinocytes in cell culture [2, 3]. This suggests that elevated 14-3-3*σ* protein levels inhibit clonal expansion of cells within the epidermal basal layer and support keratinocyte differentiation.

The p63 protein is a master regulator of epidermal morphogenesis and is exclusively expressed in the basal cell layer. *p63*-null mice lack suprabasal layers and do not express differentiation markers [4]. ΔNp63α, an alternative p63 splice variant, transcriptionally represses *14-3-3σ* expression [5]. Thus, p63 and 14-3-3*σ* exercise opposite functions in the epidermis. In line with this scenario, it has been reported that 14-3-3σ binds to ΔNp63α upon DNA damage and sequesters ΔNp63α to the cytoplasm to facilitate its proteasomal degradation [6]. Thereby, an elevated 14-3-3*σ* expression could prevent expansion of the basal keratinocyte layer.

In addition, many studies have demonstrated that *14-3-3σ* expression is deregulated or lost in several types of human epithelial cancers [7, 8]. Silencing of the *14-3-3σ* gene by CpG-methylation also frequently occurs in breast cancer [9]. Hence, loss of 14-3-3σ could be a necessary step for the initiation and/or progression of epithelial tumorigenesis.

14-3-3σ has been characterized as a p53-target genes that is sufficient and necessary to mediate a G_2_/M-arrest after DNA damage [10, 11]. 14-3-3σ proteins only form homodimers and have a distinct repertoire of ligand interactions when compared to the six other 14-3-3 isoforms expressed in human and murine cells [12, 13]. 14-3-3σ dimers bind to a large number of ligands *via* phosphorylated serine/threonine residues and thereby presumably affect a plethora of cellular processes [14]. Numerous studies based on cellular analyses have implicated 14-3-3σ in the regulation of diverse processes, such as cell cycle progression, signaling, differentiation, apoptosis and metabolism [7, 15–17]. However, relatively few studies of the organismal function and relevance of 14-3-3σ employing genetically engineered mouse models have been reported. By studying a mammary epithelial-specific *14-3-3σ* knock-out mouse it was shown that *14-3-3σ*-deficient mammary epithelial cells lose their polarity and show increased proliferation [18]. Amplification and elevated expression of the *erbB2* proto-oncogene is associated with poor outcome in patients with breast cancer [19]. It has been shown that the Cre-mediated deletion of *14-3-3σ* in an *erbB2*-driven breast cancer mouse model enhances tumor initiation and metastases [20]. Additionally, ectopic *14-3-3σ* expression reduces the metastatic capacity of a human breast cancer cell line in a xenograft mouse model [21]. Therefore, 14-3-3σ shows tumor suppressive capacities *in vivo*.

The *repeated epilation* (*ER*) mouse model has been used multiple times for studying the role of 14-3-3σ in the epidermis. *ER* mutant mice harbor a single nucleotide insertion in the *14-3-3σ* gene causing a frame shift which leads to the expression of a C-terminally truncated 14-3-3σ protein that lacks the nuclear export signal and several phosphopeptide-binding residues [22, 23]. Homozygous *ER* mutant mice (*ER/ER*) die at birth as a result of respiratory stress [22–24]: The oral cavity is fused, limbs and tail are shortened, defined digits and nails are lacking. Their epidermis is strongly hyper-proliferative and thickened accompanied with failures in terminal differentiation. The hair follicles are underdeveloped and sparse. Heterozygous *ER* mutant mice *(+/ER*) are viable. However, at postnatal day 7 they display a hyper-proliferative epidermis, however not as pronounced as observed in *ER/ER* mutant mice at embryonic day E18.5. Furthermore, *+/ER* mutant mice show defects in hair shaft differentiation, resulting in destruction of the hair shaft and a cyclic hair loss [25]. Studies analyzing this hair defect showed that the heterozygous *ER*-mutation in *14-3-3σ* specifically affects the club hair retention [26] and causes severe defects in hair shaft differentiation during morphogenesis and abnormal cycling of the hair follicle stem cells in the bulge [25]. In addition, heterozygous *ER*-mice show an extensive hyper-proliferation of the epidermis followed by the development of multiple papillomas and squamous cell carcinoma at the age of 6 month [27]. *ER*-mice display increased levels of p63 in the epidermal suprabasal layers, suggesting that truncated 14-3-3σ fails to block the transcription of *TP63* which may contribute to hyper-proliferation of the epidermis [28]. In addition, Yap1, an essential factor of the Hippo pathway controlling organ size and tissue homeostasis [29], is expressed in the epidermal suprabasal layers of *ER*-mice [30]. In keratinocytes derived from *ER* mice truncated 14-3-3σ fails to bind and sequester Yap1 in the cytoplasm, suggesting that 14-3-3σ inhibits Yap1-dependent gene expression to control epidermal proliferation and differentiation.

To date the *in vivo* role of 14-3-3σ expression in epidermal homeostasis and tumorigenesis has been mainly inferred from results obtained with *ER*-mice. However, truncation of the 14-3-3σ protein in *ER*-mice may not be equivalent to deletion and therefore complete inactivation of 14-3-3σ function.

In this study we provide evidence that the *ER* phenotype may rather result from a gain of function mutation since we determined that 14-3-3σ deficiency does not affect homeostasis of normal epidermal tissues and does not result in perinatal lethality seen in *ER/ER* mice. Nonetheless, loss of 14-3-3σ sensitizes mice to chemically-induced skin carcinogenesis. Therefore, 14-3-3σ may indeed represent a mediator of tumor suppression in the skin.

## RESULTS AND DISCUSSION

### *14-3-3σ*-deficient mice display disorganized hair but lack obvious epidermal defects

**Figure 1:**
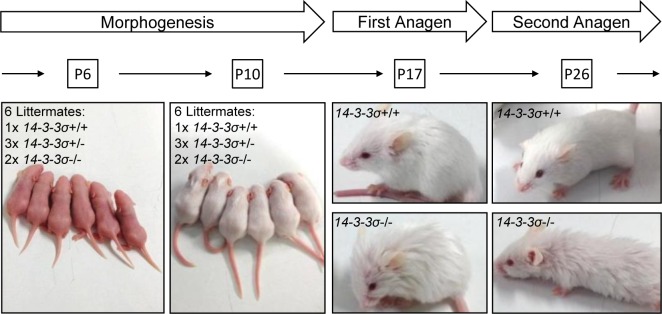
*14-3-3σ* knock-out mice show a disorganized fur from postnatal day 17 onwards Heterozygous *14-3-3σ* knock-out mice were intercrossed and pictures of the offspring with the indicated genotypes were taken at the indicated time-points.

**Figure 2:**
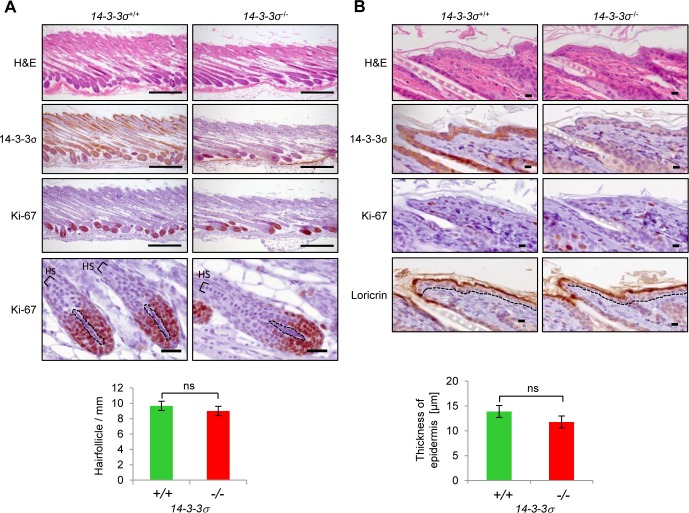
Comparison of back skin morphology in *14-3-3σ*^**+/+**^ and *14-3-3σ*^**−/−**^ mice Back skins from wild-type and *14-3-3σ* knock-out mice were resected at postnatal day 17, paraffin embedded, vertically sectioned and stained with haematoxilin/eosin (H&E, upper panels) or subjected to immunohistochemistry with 14-3-3σ, Ki-67, and Loricrin specific antibodies. **A.** Hair follicle density (scale bar = 300 μm) and hair shaft (HS) morphology (scale bar = 30 μm) are visible. The dashed line indicates the dermal papilla. Bar-chart: The average number of hair follicles per millimeter skin section was evaluated (*n* = 3). **B.** A higher magnification of the interfollicular epidermis and the underlying dermis is shown (scale bar = 10 μm). The dashed line indicates the border between epidermis and dermis. The Loricrin staining shows the outermost layer of the epidermis. Bar-chart: The average epidermal thickness was evaluated (*n* = 3).

In order to analyze whether the reported phenotypes of *ER*-mice are caused by the expression of the truncated 14-3-3σ protein or are due to 14-3-3σ loss of function, we generated mice with a floxed *14-3-3σ* allele and inactivated the *14-3-3σ* gene in the germ-line by crossing these with *deleter-Cre* mice (see also Supplemental Figure S1). By further back-crossing we obtained *14-3-3σ*-deficient mice with an FVB background. FVB mice are prone to develop tumors of the skin when subjected to chemical carcinogenesis [31]. Mice with heterozygous (*14-3-3σ*^+/−^) and homozygous (*14-3-3σ*^−/−^) deletion of 14-3-3σ were viable, fertile and born at the normal Mendelian ratio, had a normal life-span and did not display an increase in tumor formation (data not shown). *14-3-3σ*^+/−^ mice were indistinguishable from their wild-type littermates. However, *14-3-3σ*^−/−^ mice showed a disorganized fur beginning around postnatal day 17 (Figure 1). Nonetheless, the histological analysis of skin from postnatal day 17 animals did not reveal obvious alterations in hair follicle density or hair shaft shape (Figure 2). Immunohistochemical detection of Loricrin indicated no difference in epidermal thickness either. Ki-67 staining did not reveal differences in proliferation of keratinocytes between the genotypes. Therefore, *14-3-3σ*^−/−^ mice and *14-3-3σ*^+/^*^ER^* mutant mice have at least one phenotype in common: a disorganized fur onwards from approximately 2 weeks after birth. However, other phenotypes characteristic for *14-3-3σ*^+/ER^ mice were not observed in *14-3-3σ*-deficient mice: the frequency of Ki-67-positive hair follicle matrix cells (Figure 2A) or basal epidermal cells (Figure 2B) was unchanged. Additionally, the number of hair follicles and the epidermal thickness showed no differences in 14-3-3σ^−/−^ tissues (Figure 2). Similar results were obtained after tissue specific *14-3-3σ* knockout in the epidermis of mice with a the C57BL/6J background (Supplementary Figure S2A). This suggested that the epidermal hyper-proliferation observed in *14-3-3σ*^+/ER^ mice is caused by the expression of the truncated 14-3-3σ protein, which acquired additional functions and presumably an altered ligand specificity, and not by the mere loss of 14-3-3σ function. Notably, p63 and YAP1 have been shown to functionally interact with the ER mutant of 14-3-3σ in murine epidermis [28, 30]. However, we could not detect an effect of *14-3-3σ* loss on the localization and expression level of p63 (Supplemental Figure 2B). In addition, we were unable to detect a functional or direct interaction or colocalization of 14-3-3σ with YAP1 in cells *ex vivo* (data not shown). Also YAP1 expression and localization in chemically induced skin papillomas was not affected by deletion of *14-3-3σ* in mice (data not shown). The expression of a truncated ER 14-3-3σ mutant may therefore indirectly promote the deregulation of p63 and YAP1, whereas complete loss of 14-3-3σ function does not affect the function and expression of p63 or YAP1. This divergence could, at least in part, explain the phenotypic differences between the *ER* mice and *14-3-3σ* knock-out mice.

### Unchanged frequency of spontaneous epidermal tumors in *14-3-3σ* deficient mice

**Figure 3:**
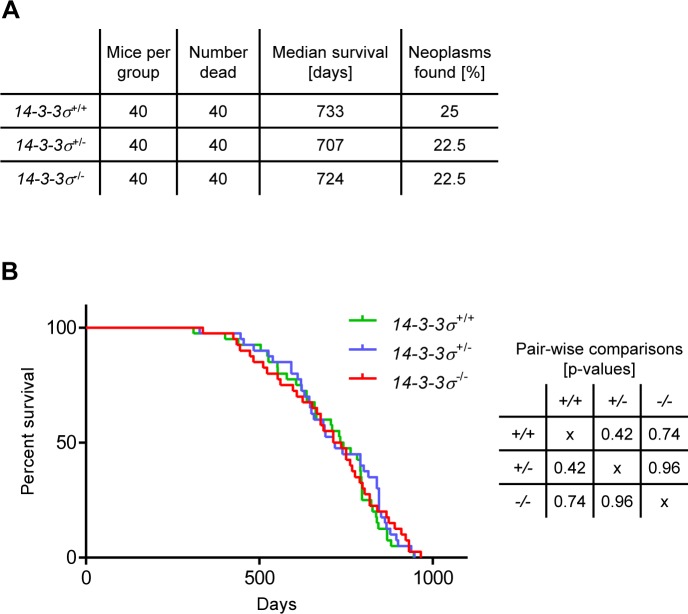
Survival analysis of mice with varying *14-3-3σ* genotype **A.** Experimental groups (20 female and 20 male mice per group) and summary of results. **B.** Kaplan-Meier curve (*n* = 40) and p-values of the curve comparisons calculated using the GraphPad Prism5 software.

To exclude the possibility that mice lacking *14-3-3σ* exhibit an attenuated epidermal hyper-proliferation phenotype similar to that of *14-3-3σ*^+/ER^ mice, we analyzed the long-term consequences of *14-3-3σ* deficiency in mice. *14-3-3σ*^+/+^, *14-3-3σ*^+/−^ and *14-3-3σ*^−/−^ (40 mice / genotype) mice were maintained for a prolonged time period (up to 32 months) and were inspected twice a week for the presence of neoplastic lesions or other pathologies. Mice with tumors larger than 2 cm in diameter or with other signs of illness were euthanized. Dead mice were analyzed by necropsy. Tumor burden of dead mice was recorded (Figure 3A). Time points of death were noted and used for calculation of the survival probability of each genotype (Figure 3A, 3B). No significant difference in survival between wild-type, heterozygous and knockout *14-3-3σ* mice was found. In addition, *14-3-3σ* knockout mice did not show a significantly increased risk of spontaneous tumor development (Figure 3A, 3B). Furthermore, the tumor burden was independent from the genotype (Figure 3A). These results show that *14-3-3σ* deficiency is not sufficient to initiate spontaneous skin carcinogenesis. Therefore, the development of multiple papillomas and squamous cell carcinomas observed in *14-3-3σ*^+/ER^ mice [27] is presumably not caused by the loss of 14-3-3σ function but rather by the expression of a truncated 14-3-3σ protein, which presumably acquired additional, non-physiologic properties.

### *14-3-3σ* deficiency increases number and size of DMBA/TPA induced papillomata

**Figure 4:**
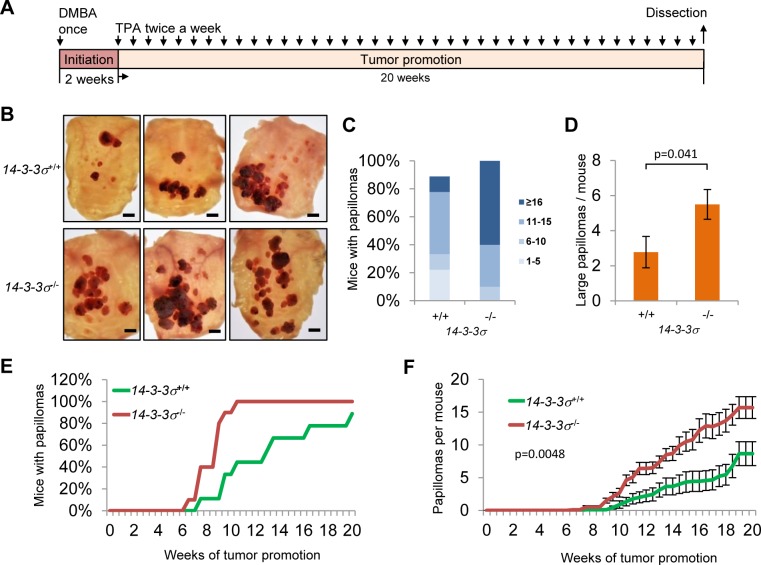
Effect of *14-3-3σ*-deficiency on the number and size of DMBA/TPA-induced skin tumors **A.** *14-3-3σ*^+/+^; *n* = 9 and *14-3-3σ*^−/−^; *n* = 10 male mice were treated once with 100 nmol DMBA. After 2 weeks all mice were treated twice a week with 13.6 nmol TPA for 20 weeks. **B.** Overview of representative back skin resections (scale bar = 5 mm). **C.** Cumulative percentage of mice carrying the indicated numbers of tumors. **D.** Average numbers of large tumors (surface ≥ 15 mm^2^). Results are the mean ± s.e.m.; *p* = 0.041 by two-sided t test. **E.** Percentage of mice with detectable papillomas during the period of tumor promotion. **F.** Average number of tumors during the period of tumor promotion. Results are the mean ± s.e.m. with *p* = 0.0048 by Mann-Whitney *U* test.

**Figure 5:**
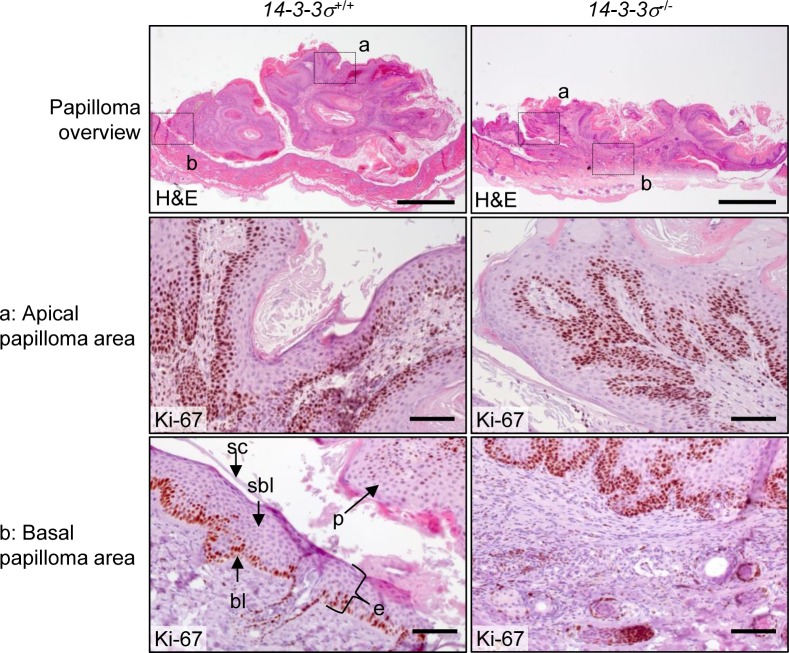
Morphology of DMBA/TPA-induced epidermal tumors in mice with varying *14-3-3σ* genotype Skin sections containing papillomas from *14-3-3σ*^+/+^ and *14-3-3σ*^−/−^ were stained with haematoxilin/eosin and probed for Ki-67. The upper panel shows an overview of the whole papilloma/skin area (scale bar = 1 mm). The dashed boxes in the overview indicate the magnified areas of **a.** an apical tumor region, **b.** a tumor attachment site shown in the lower panels (scale bar = 100 μm).

The *14-3-3σ* gene is frequently silenced in basal cell and squamous cell carcinoma [8, 32, 33]. Nevertheless, the pathophysiological relevance of *14-3-3σ* inactivation for epithelial skin cancer formation is largely unknown. In order to study the effect of *14-3-3σ* inactivation on the onset, frequency and progression of chemically-induced papillomas of the skin, which ultimately progress to squamous cell carcinomas (SCC), we employed a well-established two-stage model of chemical skin-carcinogenesis which is based on DMBA/TPA treatments [34]. The back skin of male wild-type and *14-3-3σ* knockout mice (7 to 9 weeks old) was treated in the pause phase of the hair cycle with a single dose of 7.12-dimethylbenz(a)anthracene (DMBA) for tumor initiation (Figure 4A). Two weeks later, both groups were treated twice a week with 12-O-tetradecanoylphorbol-13 acetate (TPA) for a total period of 20 weeks to achieve tumor promotion. The time points of tumor occurrence, number and size were quantified throughout the complete treatment period (Figure 4C-4F). After 20 weeks the back skin was resected (Figure 4B) and the number (4C) and size (4D) of tumors present in these specimen was determined. Subsequently, tumors were analyzed histologically (Figure 5). ~7 weeks after the first TPA administration the first skin tumors (≥1 mm diameter) occurred in both groups (Figure 4E). Over the subsequent 13 weeks of tumor promotion, the number of mice with tumors (Figure 4E) and the number of tumors per mouse (Figure 4F) increased significantly faster in *14-3-3σ* -deficient mice. By the end of the TPA treatments, 100% of the *14-3-3σ*-deficient and 89% of the wild-type mice had developed numerous tumors. 60% of the *14-3-3σ* deficient and only 11% of the wild-type mice had 16 or more tumors (Figure 4C). Counting of tumors with an area of ≥15mm^2^ revealed that *14-3-3σ* wild-type mice developed on average 2.8, whereas *14-3-3σ* knock-out mice displayed 5.5 papilloma of this size (Figure 4D). Taken together, *14-3-3σ*-deficient mice developed 1.8-fold more papillomas per mouse in total and 1.98-fold more larger tumors (≥15mm^2^) per mouse than wild-type mice (Figure 4F, 4D). Further examination revealed that all induced tumors from wild-type and *14-3-3σ* knock-out mice projected outwardly, with a cauliflower-like shape, which is a characteristic of benign papillomas (see example in Figure 5). Next we obtained tissues sections containing tumors of different sizes from wild-type and *14-3-3σ*-deficient mice and analyzed them microscopically after haematoxilin/eosin staining and by immunohistochemistry (Figure 5). Like we already had deduced from the external examination of the tumors (Figure 4B), inspection of the histological sections confirmed a tumor growth directed towards the outer side of the epidermis (Figure 5). The tumors resemble the architecture of normal skin with a basal layer, stratified squamous epithelium and an outer keratinized layer (Figure 5A). No invasion was observed (Figure 5B). Taken together our results show that *14-3-3σ* deficiency significantly increases the number and size of DMBA/TPA-induced papillomas. Thus, 14-3-3σ fulfills a tumor suppressive function *in vivo*, which presumably prevents the initiation and growth of epidermal tumors. This is in line with the timing of epigenetic inactivation of *14-3-3σ* in tumors, which has been described to occur in the early phases of tumor progression [35].

## MATERIALS AND METHODS

### Generation and handling of mice

The detailed description of the generation of *14-3-3σ* deficient mice can be found in supplementary material and methods (Figure S1). *14-3-3σ*^−/−^ mice were transferred into an FVB background by backcrossing for at least 6 generations with FVB wild-type mice. For the experiments, FVB littermates of the genotypes *14-3-3σ*^+/+^, *14-3-3σ*^+/−^ and *14-3-3σ*^−/−^ were generated by intercrossing of FVB *14-3-3σ*^+/−^ mice. All mice were maintained in the animal facility at the Pathology Institute of the Ludwig-Maximilians-University Munich in individually ventilated cages (IVC). All animal experiments were approved by the Regierung von Oberbayern (AZ: 55.2-1-54-2532-11-13).

### Genotyping PCR

Genomic DNA was obtained by overnight digest of ear punches in lysis buffer (50 mM KCl, 1.5 mM MgCl_2_, 10 mM Tris pH8.5, 0.01% gelatin, 0.45% NP40, 0.45% Tween 20, 100 μg/ml proteinase K). PCR was performed for 35 cycles with an annealing temperature of 65°C. Primers for the detection of the *14-3-3σ* wild-type and floxed alleles (forward: 5′-CAC TAC CGT GGT CTT CCC TAA CTT GAT G-3′; reverse: 5′-TCC CAG GAA GCA GAT GGG ATT TCT GTC C-3′) and the *14-3-3σ* knock-out allele (reverse: 5′-AGG CAC TAT GCC CCT GCC TCA GAT-3′) were added in the same ratio to the reaction mix and PCR products were resolved on 2% agarose or 8% polyacrylamide gels. The PCR-products were 106 bp (wild-type allele), 159 bp (floxed allele), and 183 bp (*14-3-3σ* knock-out allele).

### Determination of hair follicle numbers and epidermal thickness

Dorsal skin from male *14-3-3σ*^+/+^ and *14-3-3σ*^−/−^ FVB littermates was dissected, fixed in 4% formalin, paraffin embedded, vertically sectioned at 5 μm distances, stained with haematoxilin and eosin (H&E), mounted and analyzed by light microscopy. Hair follicles were counted over an epidermis length of 1 mm from 3 individuals per genotype. In addition, the epidermal thickness was measured at 3 randomly chosen positions of the epidermis of 350 μm length using the AxioVison Rel4.8 software. Average numbers, standard deviation and significance were calculated using a Student's test (two-sided; *n* = 3).

### Survival probability

40 mice (20 male, 20 female) per genotype (*14-3-3σ*^+/+^, *14-3-3σ*^+/−^, *14-3-3σ*^−/−^) with a FVB background were maintained and monitored over a period of more than 2 years. Moribund mice were euthanized and skin sections were analyzed immunohistochemically. Tumor burden [No = 0, Yes = 1] and age at death [in days] was documented. Survival probability was calculated according to Kaplan-Meier using GraphPad Prism5 software. Additionally, the percentage of mice with tumors was calculated.

### Chemically-induced skin carcinogenesis

The back skin of male *14-3-3σ*^+/+^ (*n* = 9) and *14-3-3σ*^−/−^ FVB (*n* = 10) littermates (7-9 weeks of age) was shaved 2 days prior to tumor initiation. Tumors were initiated with a single topical application of DMBA (Sigma-Aldrich, Munich, Germany, order no. 40567, 100 nmol in 50 μl methanol) to the back skins of the mice. Two weeks later, tumor promotion was achieved by topical application of TPA (Sigma-Aldrich, Munich, Germany, order no. P1585, 13.6 nmol in 200 μl acetone) twice weekly for 20 weeks. Tumor incidence (percentage of tumor-bearing mice) and multiplicity (papillomas per mouse) were recorded twice weekly throughout the experiment. Pictures of dissected back skin were taken and used to determine the sizes of papillomas [mm^2^] with the ImageJ software.

### Immunohistochemistry

Tissues were fixed in 4% formalin and embedded in paraffin. 5 μm sections were prepared, deparaffinized in xylene and rehydrated in serial ethanol dilutions. Antigen retrieval was carried out by boiling in DAKO citrate buffer (pH 6.0) twice for 15 minutes in a microwave oven at 750 Watts. After quenching endogenous peroxidase activity by 10 min. exposure to 7.5% H_2_O_2_ solution, tissue sections were blocked in either 10% goat or rabbit serum, or 10% BSA for 30 min. at room temperature depending on the origin of the primary antibody. Primary antibodies were used at 1:20 dilution for anti-14-3-3σ (C-18, goat polyclonal anti-mouse, Santa Cruz), 1:500 for anti-Ki67 (D3B5, rabbit monoclonal anti-mouse, Cell Signaling), 1:500 for anti-Loricrin (AF62, rabbit polyclonal anti-mouse, Covance) and 1:100 for anti-p63 (4A4, Dako, supplementary data). Biotinylated secondary antibodies and streptavidin/HRP complexes were used according to the provided manual (Vectastain kit, Vector Labs). Sections were treated with DAB chromogenic substrate (DAKO), counterstained with haematoxilin and mounted in xylene-based mounting medium.

## SUPPLEMENTARY MATERIAL



## Abbreviations

ER: repeated epilation
DMBA: 7.12-dimethylbenz(a)anthracene
TPA: 12-O-tetradecanoylphorbol-13 acetate
