# Supplementary material for: Deletion of 14-3-3σ sensitizes mice to DMBA/TPA-induced papillomatosis
Source: Oncotarget. 2016 Jul 7;7(30):46862–70. doi: 10.18632/oncotarget.10478 (PMC5216908; doi:10.18632/oncotarget.10478)
Supplement: Supplementary file 1 [file oncotarget-07-46862-s001.pdf]

# Deletion of *14-3-3σ* sensitizes mice to DMBA/TPA-induced papillomatosis

**Supplementary Material**

**Mouse strains and husbandry**

Blastocysts isolated from C57BL/6 mice were used for injection of *14-3-3σ*$^{+/fl}$ ES cells derived from Sv129 mice. By further crossing of chimeras heterozygous and homozygous out-bred animals were obtained (mixed C57BL/6 x Sv129 background). To generate mice deficient for *14-3-3σ* in epidermal tissues we used Keratin5-Cre transgenic mice (*K5-Cre*$^{+/-}$; *14-3-3σ*$^{fl/fl}$) . In order to generate germ-line deficient *14-3-3σ* mice (*14-3-3σ*$^{-/-}$) we used a deleting-Cre line (*CMV-Cre*$^{+/-}$). Inbred strains and generated mutants were maintained in a barrier facility with specific pathogen-free conditions at the animal facility of the MPI of Biochemistry. Subsequently, mice were kept in individually ventilated cages (IVC) at the Ruhr-University Bochum and the Ludwig-Maximilians-University, Munich.

**Construction of targeting vector**

A genomic DNA fragment of 4.5 kb size was isolated from a BAC clone containing the mouse *14-3-3σ* locus (derived from 129/Sv strain) by BamHI and BglII digestion and cloned into a pBlueScript-KS vector (Stratagene). A short DNA linker (5’-AGATCTGGACGCGTGGGACTAGTGCGGCCGC-3’) was ligated between the BglII and NotI sites of this vector creating additional MluI and SpeI sites. The 3’ homologous arm was amplified by PCR using primers #438 (5’-GGGACGCGTGAGCCGAGGGGTGCAATCTGAGGCAGGGG-3’, introduces a MluI site) and #437 (5’-ACGGCTCTGTGGGTCACAAACATAGCAGAAA-3’) and BAC DNA as a template. After digestion with MluI and SpeI, this 3.5-kb fragment was inserted downstream

of the 5’ homologous arm. The region containing proximal promoter and the only exon of *14-3-3*$\sigma$ was amplified by PCR using primers #430 = 5’-GGGGAGCAAACTCCATACACACTAGCAGGG-3’ and #431 = 5’-AGAAGGGTAGCGCGGCCGACGGGGCGGGAGG-3’, digested by BglII and cloned into BclI site of shuttle vector between two modified loxP sites [1]. After sequencing, the floxed cassette was excised by BglII digestion and inserted into the BglII site of the targeting vector in sense orientation. A selection marker comprising a PGK promoter, neomycin resistance gene and polyadenylation signal flanked by frt sites (Flp recombinase target sites) was introduced into I-SceI restriction site. The functionality of loxP sites was tested by *in vitro* treatment with Cre recombinase (NEB) and restriction analysis.

**Generation of mutant ES cell clones and chimeric mice**

The R1 129/Sv ES cell line was cultured on a feeder layer of γ-irradiated embryonic fibroblast cells (derived from mice carrying a neomycin resistance gene) in Dulbecco's modified Eagle medium supplemented with 20% heat-inactivated fetal calf serum (GIBCO), 2 mM L-glutamine, 0.1 mM ß-mercaptoethanol (Sigma) and non-essential amino acids (GIBCO) in the presence of 1000 U/ml LIF (ESGRO from GIBCO). $4x10^7$ R1 ES cells were electroporated with 100 µg of NotI-linearized targeting vector using a Bio-Rad Gene Pulser set at 800 V and 3 µF. After 24 hrs without selection, G418 was added to the culture medium at the concentration 500 µg/ml. After another 7 days, 360 colonies were picked into 24-well plates and expanded. Half of the cells from 24-well dishes were frozen, and the other half was used to isolate DNA for Southern blot analysis. Clones positive for homologous recombination as determined by Southern blot analysis were thawed and expanded. Blastocysts were isolated at day 3.5 p.c. from C57BL/6 mice, injected with 15 ES cells (four different ES clones were used in total) and transferred into the uterus of pseudo-pregnant C57BL/6 recipient mice. The degree of chimerism was assessed by agouti coat color. Chimeric males were mated with C57BL/6 females to test for germ-line transmission.

**Southern blot analysis**

Genomic DNA was prepared from ES cell clones by SDS/proteinase K digest followed by isopropanol precipitation. 20 µl (10% of DNA recovered from a well of 24-well plate) were digested in total volume 30 µl with HindIII or BamHI enzymes and separated by gel-electrophoresis using 0.7% agarose. Using a MegaPrime Kit (Amersham) the $^{32}P$-labeled external probe was generated by random primer labeling of a 0.6-kb genomic BclI-HindIII fragment of the PCR product amplified with primers #437 and #438. A BamHI-KpnI genomic fragment from the 5‘ homologous arm was used as an internal probe for confirming the integration of the 5‘ loxP site. After capillary transfer of DNA to a nylon membrane (Hybond) and UV crosslinking, blots were hybridized in QuickHyb solution (Stratagene) for 2 hours at 68°C. Radioactive signals were detected using a Phosphor-imager (Fuji).

**Immunohistochemistry**

Tissues were fixed in buffered formalin solution and embedded in paraffin. 6 µm sections were deparaffinized in xylene and rehydrated in serial ethanol dilutions. Antigen retrieval was carried out by boiling in citrate buffer (pH 6.0) twice for 10 minutes in a microwave oven. After quenching endogenous peroxidase activity by 10 min exposure to 7.5% $H_2O_2$ solution, the tissue-section was blocked in 10% horse serum for 30 min. Primary antibodies were used at 1:500 for anti-Ki67 (NeoMarkers) and 1:100 for anti-p63 (4A4, Dako). Biotinylated secondary horse antibodies and streptavidin/HRP complexes were used according to the provided manual (Vectastain kit, Vector Labs). Sections were treated with AEC (Ki67) or DAB (p63) chromogenic substrate, counterstained with haematoxilin and mounted in Aquatex (Merk) on Entalan (Merk) medium.

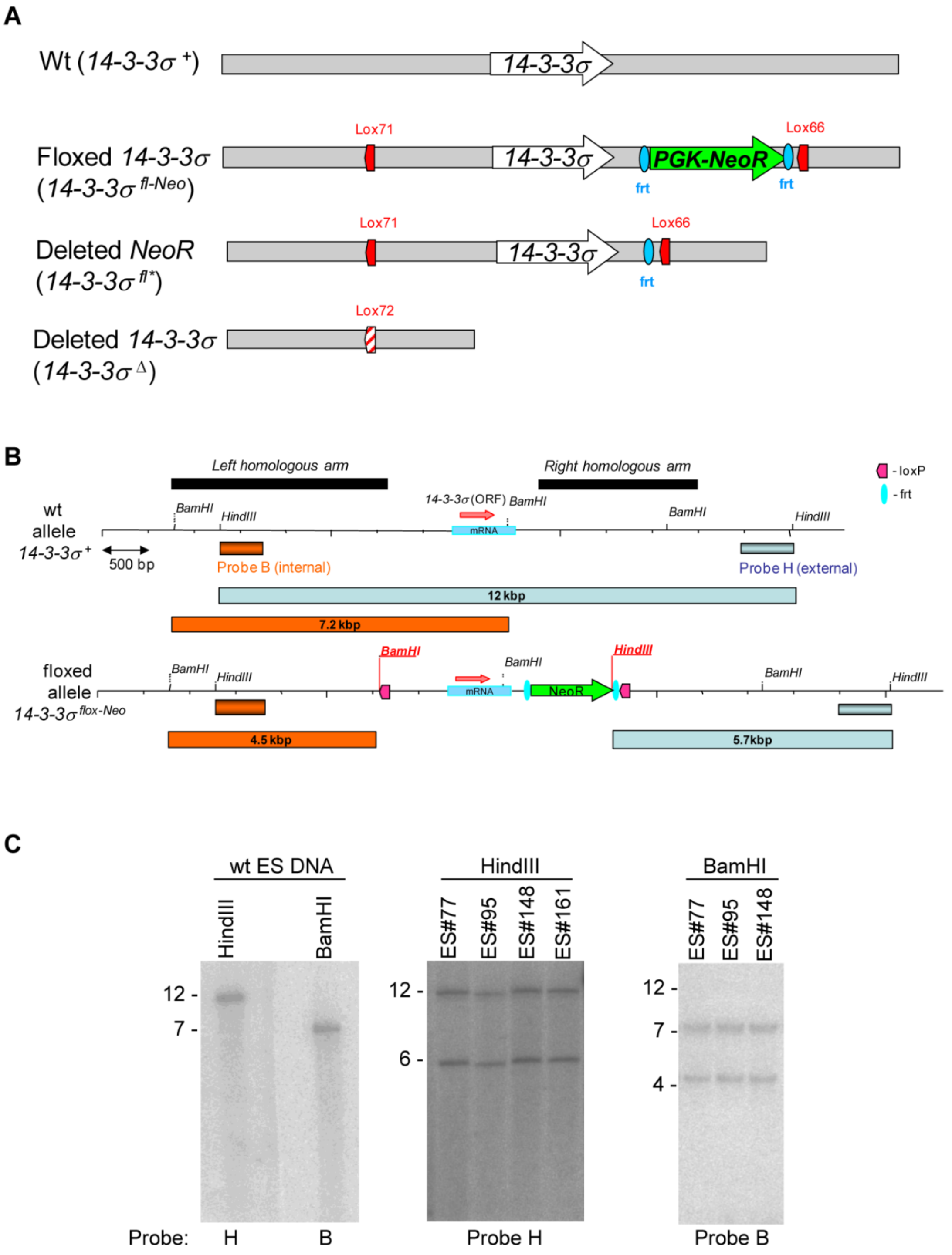


**Supplemental Figure 1:**

**Generation of conditional *14-3-3σ* knock-out mice by homologous recombination.**

**(A)** Knock-out strategy for conditional inactivation of *14-3-3σ* and allele nomenclature.

**(B)** Strategy for detection of targeted integration by Southern blot analysis. **(C)** Southern blot analysis of mouse ES cells. The numbers indicate the size of marker DNA fragments in kilo base-pairs.

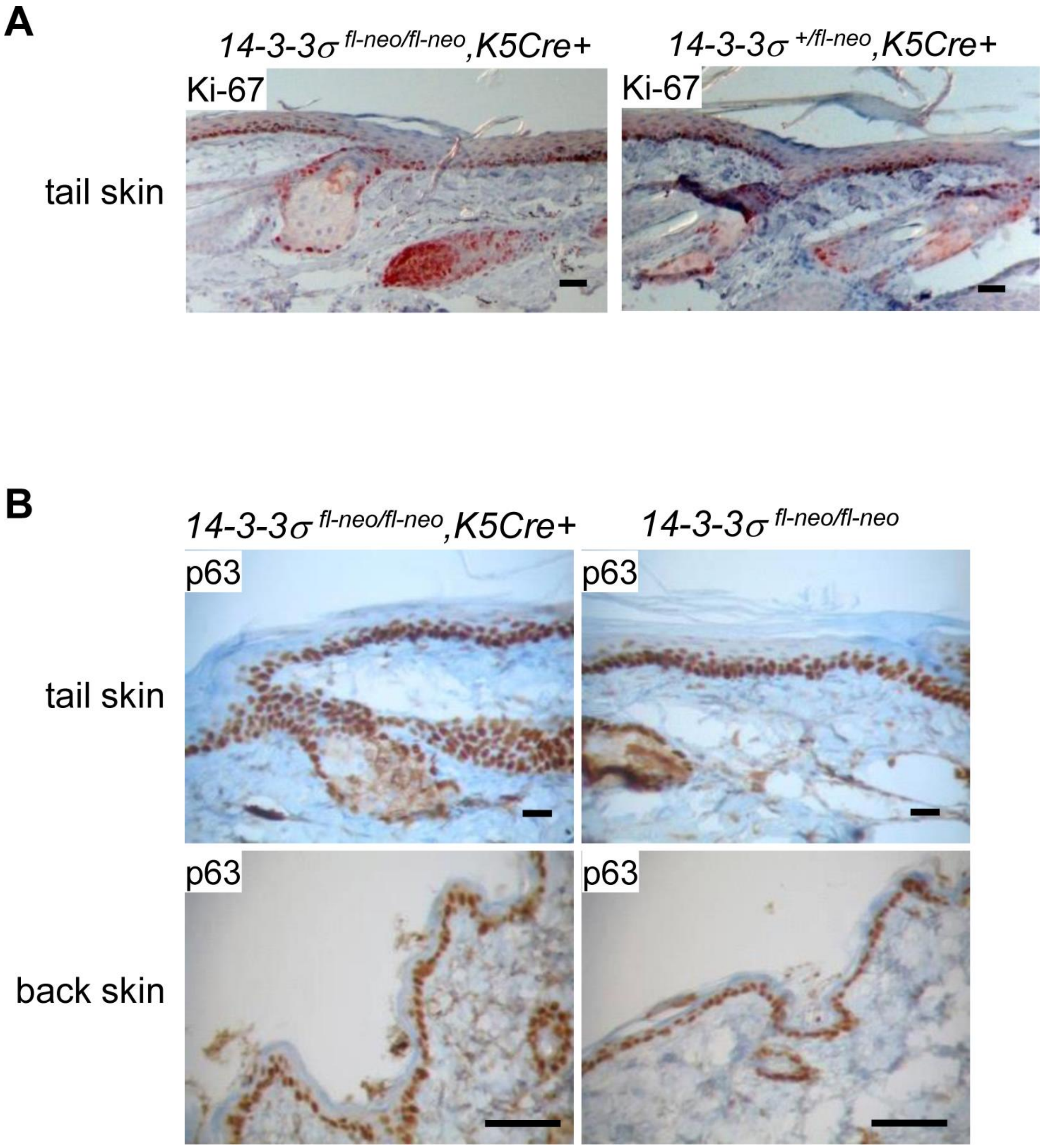


**Supplemental Figure 2:**

**Analysis of the effect of *14-3-3σ* deletion on epidermal proliferation and differentiation.**

Representative staining results with FFPE-sections derived from 10 weeks old mice with the indicated genotypes. **(A)** Detection of proliferating cells using Ki67-specific antibodies (red nuclear staining). Magnification: 400x ; scale bar size = 20 µm. **(B)** A mouse monoclonal antibody (4A4 clone) detecting all isoforms of p63 was used for immunohistochemical analysis (brown nuclear staining). Magnification: 400x ; ; scale bar size = 20 µm (top panel) and 50 µM (bottom panel).

**Supplemental References**


1. Arakawa H, Lodygin D and Buerstedde JM. Mutant loxP vectors for selectable marker recycle and conditional knock-outs. BMC biotechnology. 2001; 1:7.
